# Utility of bronchoalveolar lavage in diagnosing respiratory tract infections in patients with hematological malignancies: are invasive diagnostics still needed?

**DOI:** 10.1080/03009734.2016.1237595

**Published:** 2016-10-14

**Authors:** Tobias Svensson, Kristina Lamberg Lundström, Martin Höglund, Honar Cherif

**Affiliations:** a Department of Medical Sciences, Section of Hematology, Uppsala University, Uppsala, Sweden;; b Department of Medical Sciences, Section of Pulmonology, Uppsala University Hospital, Uppsala, Sweden

**Keywords:** Aspergillosis, bronchoalveolar lavage, hematological malignancies, immunodeficiency, invasive fungal disease, neutropenia, Pneumocystis jirovecii pneumonia, pulmonary infiltrates

## Abstract

**Background:**

Patients treated for hematological malignancies have an increased risk of serious infections. Diagnosis and prompt initiation of therapy are essential. Bronchoalveolar lavage (BAL) is a well-established investigation for identifying the cause of pulmonary infiltrates in immunocompromised patients. The aim of the study was to determine the diagnostic yield of BAL in patients treated for hematological malignancies and how often it contributed to a modification of the anti-infectious therapy.

**Methods:**

We reviewed records from 151 consecutive BAL procedures in 133 adult patients with hematological malignancies, treated at a tertiary hematology unit from 2004 to 2013. Extensive microbiological work-ups on BAL samples had been performed according to a standardized protocol.

**Results:**

A microbiological finding causing the infectious episode could be identified in 59 (39%) cases. In 44 (29%) of the cases, results from BAL had an impact on clinical management either by contributing to a specific diagnosis (25%) or by leading to cessation of ongoing microbiological therapy. The most common diagnoses were invasive pulmonary aspergillosis (IPA) and *Pneumocystis jirovecii* pneumonia (PJP). Diagnoses of IPA and PJP were based on results from BAL in 65% and 93% of cases, respectively. Several microbiological tests on BAL samples rendered no positive results. Complications were few and mainly mild.

**Conclusion:**

BAL is still important for either verifying or excluding some of the most important respiratory tract pathogens in patients with hematological malignancies, particularly IPA and PJP. Standardized procedures for BAL sampling should be continually revised to exclude unnecessary microbiological tests.

## Introduction

Patients treated for hematological malignancies, in particular those undergoing allogeneic (allo-) hematopoietic stem cell transplantation (HSCT), have an increased risk of serious infections. Neutropenia, decreased cellular immunity, hypogammaglobulinemia, chemotherapy-induced damage to mucosal barriers, and the frequent use of central venous lines predispose to such infections (1–4). The panorama of pathogenic micro-organisms affecting this patient group is broad and includes such opportunistic micro-organisms as *Aspergillus* spp., *Candida* spp., *Mycobacterium* spp., *Pneumocystis jirovecii*, and respiratory viruses (1–4). In patients with severe neutropenia related to hematological disease, or its treatment, 40% to 60% develop pulmonary infiltrates (1,5,6), and pneumonia is the primary cause of mortality not directly related to the hematological malignancy itself. However, in the majority of these cases the causative pathogen remains undetected (4,7,8).

Bronchoalveolar lavage (BAL) is a well-established method for identifying the cause of pulmonary infiltrates not evident by conventional non-invasive measures. The diagnostic yield from BAL in patients with hematological malignancies varies from 15% to 90% in different studies (1,5–7,9–11); the earlier the investigation, the greater the likelihood of finding the causative pathogen (3,9). Infrequent but serious complications are major bleeding, pneumothorax, and deterioration to respiratory failure. Procedure-related mortality is extremely rare (1,3,7,9,12,13). Non-invasive methods for diagnosing invasive fungal disease (IFD) are under constant improvement. Thus, laboratory tests to detect *Aspergillus* galactomannan (GM), 1,3-beta-D-glucan (BG), or polymerase chain reaction (PCR)-based methods (2,14–18), although not yet standardized and validated, as well as radiological methods could facilitate the diagnosis (14).

The major aim of the present study was to determine the diagnostic yield of BAL in consecutive adult patients treated in a tertiary hematology unit over a 10-year period. Further aims were to evaluate the usefulness of a standardized diagnostic procedure and its safety.

## Material and methods

### Inclusion criteria and BAL procedure

Indication for BAL was pulmonary infiltrates or fever with or without respiratory tract symptoms but no radiological findings, indicating infection but with no obvious etiology, in immunocompromised patients with hematological malignancies. Prophylactic platelet transfusions were given prior to BAL in cases of severe thrombocytopenia. BAL was performed according to a standardized procedure by a limited number of experienced specialists in the day-care unit of the Department of Pulmonary Medicine, Uppsala University Hospital. BAL samples were analyzed using a standard protocol, which was updated in 2007 and 2012 (see Appendix 1). All samples were analyzed locally with the exception of GM, PCR for *Aspergillus* spp., and tests for *Pneumocystis jirovecii*, which were sent to an external academic laboratory for investigation.

### Study design

All BAL procedures in adult patients with hematological malignancies treated at the Department of Hematology, Uppsala University Hospital from 2004 to 2013 were retrospectively reviewed in this study. Patients were identified through the local bronchoscopy register. Each BAL procedure was considered as one case/patient. Patients’ records were reviewed for demographics, clinical characteristics (Table 1), radiological findings, and test results from BAL and from ‘non-invasive’ microbiological studies (Table 2). Changes in management of anti-infectious therapy following BAL and major procedure-related complications were also recorded. The study was approved by the Regional Ethic Review Board in Uppsala.

**Table 1.** Patient characteristics.

Characteristics	*n* = 151 (%)
Unique patients	*n* = 133
Age, years, median (range)	59 (19–84)
Gender	
Male	79 (59)^a^
Female	53 (41)
Diagnosis	
AML	69 (46)
B-ALL^b^	18 (12)
Lymphoma^c^	17 (11)
Myeloma	15 (10)
CLL	13 (9)
CML	8 (5)
MDS	6 (4)
MPN	8 (5)
Other	10 (7)
Deceased	
At end of study period	77^a^ (58)
Within 30 days from time of BAL	5 (3)
Within 1–6 months from time of BAL	35 (23)
Still living	56^a^ (42)
Allo-SCT	
Yes	53 (35)
No	98 (65)
Time from allo-SCT to BAL	
Within 1 month	8
Within 1–3 months	8
Later than 3 months	37
Active GVHD in allo-SCT patients	
Yes	19
No	34
Auto-SCT	
Yes	15 (10)
No	136 (90)
Time from auto-SCT to BAL	
Within 1 month	6
Within 1–3 months	0
Later than 3 months	9
Infectious symptoms at time of BAL	
Fever	103 (68)
Cough	41 (27)
Hypoxia and dyspnea	17 (11)
Fever only	11 (7)
Other	9 (6)
Time (days) from onset of infectious symptoms to time of BAL, median (range)	9 (0–371)
Platelets at time of BAL, ×10^9^/L, median (range)	61 (<5–825)
Neutropenia^d^ at time of BAL	
Yes	54 (36)
No	97 (64)
Ongoing treatment at time of BAL	
Systemic broad-spectrum antibiotics	98 (65)
Systemic broad-spectrum antimycotics	53 (35)
Antibiotics and antimycotics^e^	44 (29)
Time (days) of treatment before BAL, median (range)	
Systemic broad-spectrum antibiotics	5 (1–24)
Systemic broad-spectrum antimycotics	3 (1–48)
Pulmonary infiltrates on CT scan and/or X-ray	
Yes	139 (92)
No	12 (8)
Ongoing immunosuppressive treatment at time of BAL	
Low-dose steroids	32 (21)
High-dose steroids^f^	18 (12)
Cyclosporin	29 (19)
No	90 (60)

a Numbers calculated on 133 unique patients.

b Including Burkitt’s lymphoma and lymphoblast lymphoma.

c Including high-grade non-Hodgkin’s lymphoma, low-grade non-Hodgkin’s lymphoma, Hodgkin’s lymphoma, and T-cell lymphoma.

d Absolute neutrophil count (ANC) below 0.5 × 10^9^.

e Systemic intravenous or oral agents, excluding prophylaxis, with a wide therapeutic range.

f A daily dose of ≥20 mg prednisone (or equivalent).

**Table 2.** Impact on diagnosis and clinical management.

Impact of BAL on clinical management	*n* = 151 (%)
Diagnosis based solely on BAL	27 (18)
Diagnosis based on BAL and other diagnostic methods	11 (7)
Negative findings from BAL leading to cessation of initiated therapy	6 (4)
No impact	107 (71)
Method for establishing final diagnosis	*n* = 151 (%)
Diagnosis based solely on BAL	27 (18)
Diagnosis based on BAL and other diagnostic methods	11 (7)
Diagnosis based on other methods than BAL	21 (14)
No diagnosis established	92 (61)
Final diagnosis^a^	*n* = 157 (%)
Aspergillus pneumonia	23 (15)
Pneumocystis pneumonia	14 (9)
Other specified cause^b^	26 (17)
Pneumonia with unknown cause	85 (54)
Neutropenic fever and/or respiratory tract symptoms with unknown cause	9 (6)

a More than one diagnosis in some cases.

b Bacterial pneumonia with specified cause, invasive candidosis, Mycobacterium tuberculosis, RS-virus, CMV pneumonitis, pulmonary GVHD, bacterial sinusitis with established cause, drug reaction, heart failure, Legionella pneumonia, Rhino virus, pulmonary embolism, COP.

### Definitions

Results from a BAL sampling were considered clinically important if they altered clinical management of antibacterial, antiviral, or antifungal therapy, as recorded in the patient record by the attending physician. Invasive pulmonary aspergillosis (IPA) was defined according to the ‘Revised Definitions of Invasive Fungal Disease’ of the European Organization for Research and Treatment of Cancer/Invasive Fungal Infections Cooperative Group and the National Institute of Allergy and Infectious Diseases Mycoses Study Group (EORTC/MSG) Consensus Group (15). Complications of the procedure were considered if they occurred during the bronchoscopy or within the next 24 hours.

### Statistics

Results were analyzed using descriptive methods and presented as median and range. Categorical variables and diagnostic yields were expressed as frequencies and percentages.

## Results

### Patient characteristics

In total, 151 BAL examinations were performed in 133 different patients (median age 57 years, range 19–84) with hematological diseases from January 2004 to December 2013. In 10 patients BAL had been performed twice and in four patients three times. The most common hematological diagnoses were acute myeloid leukemia (AML) (46%), acute lymphocytic leukemia (ALL) (12%), and multiple myeloma (10%). Fifty-three patients (35%) had undergone allo-HSCT and 15 (10%) autologous HSCT prior to BAL (Table 1). The 30-day and 6-month mortality rates were 3% (5 cases) and 23% (35 cases), respectively. The overall mortality rate at the end of December 2013 was 58% (77 cases).

At the time of BAL, 54 patients (36%) had severe neutropenia (ANC <0.5 × 10^9^/L), 36 (24%) of whom had a high-risk score on the MASCC (Multinational Association for Supportive Care in Cancer) scale (16). Thrombocytopenia was common (median platelet count of 61 × 10^9^/L, range 0–825). At least one radiological chest examination had been performed in all cases shortly prior to BAL, most of which (89%) included a CT scan. Pulmonary infiltrates were observed in 139 (92%) cases. Eleven patients (7%) had fever as their only symptom. A majority, 98 patients (65%), were on empirical broad-spectrum antibiotics, and 53 (35%) patients were treated with broad-spectrum antimycotics. Median duration of pre-BAL antibiotic and antimycotic treatment was 5 (range 1–24) and 3 (range 1–48) days, respectively (Table 1).

### Findings in BAL

Most frequent positive findings were seen on general cultivation for different species of fungi (22% of performed tests), PCR for Candida (20% of performed tests), and PCR for *Pneumocystis jirovecii* and cytomegalovirus (CMV) with positive findings in 19% and 17% of the performed tests, respectively. However, the treating physician considered a majority of the findings as contaminations or otherwise clinically irrelevant not leading to any change of diagnosis or treatment. Several investigations such as PCR for Legionella, Chlamydophilia, Mycoplasma, and Pneumococci rendered no positive results. Duplicate samples obtained using a protected specimen brush (PSB) did not yield any additional diagnostic information (data not shown).

### Final diagnosis and impact of BAL on clinical management

In59 cases (39%) at least one microbiological agent that most probably had caused the infectious episode was identified. In 92 (61%) cases no etiological diagnosis was obtained, and these patients were finally diagnosed with pneumonia, febrile neutropenia, or lower respiratory tract infection of unknown cause (Table 2). The most common final diagnosis was IPA (23 cases), of which 2 were classified as proven, 8 as probable, and 13 as possible. The second most frequent diagnosis (14 cases) was *Pneumocystis jirovecii* pneumonia (PJP). In 38 cases (25%), results from BAL had an impact on clinical management either by establishing or contributing to the final diagnosis. In another 6 cases (4%) negative findings from BAL led to cessation of ongoing microbiological therapy (Table 2). Importantly, the diagnosis of IPA was based on results from BAL in 15 (65%) of the cases and by other methods in 8 (35%) cases. In almost all patients (13 of 14 patients) with PJP, the diagnosis was based on findings obtained from BAL (Table 3).

**Table 3.** Methods for establishing Aspergillus pneumonia and Pneumocystis pneumonia.

Method for establishing diagnosis Aspergillus pneumonia^a^ (*n* = 23)	*n* (%)
Aspergillus infection proven	2 (9)
Aspergillus infection probable	8 (35)
Aspergillus infection possible^b^	13 (57)
Diagnosis based solely on BAL	10 (43)
Diagnosis based on BAL and other diagnostic methods	5 (22)
Diagnosis based on other methods	8 (35)
Positive antigen for Aspergillus in BAL^c^	3 (13)
Positive PCR for Aspergillus in BAL	4 (17)
Positive direct microscopy for Aspergillus in BAL	1 (4)
Positive cultivation for Aspergillus in BAL	8 (35)
Positive antigen for Aspergillus in blood^c^	9 (39)
Positive PCR for Aspergillus in blood	0 (0)
Other methods (not based on BAL) contributing to diagnosis	2 (9)
Typical X-ray finding for Aspergillus^d^	8 (35)
Method for establishing diagnosis Pneumocystis pneumonia^a^ (*n* = 14)	*n* (%)
Diagnosis based solely on BAL	11 (79)
Diagnosis based on BAL and other diagnostic methods	2 (14)
Diagnosis based on other methods	1 (7)
Positive immune morphology for *Pneumocystis jirovecii* in BAL	4 (29)
Positive PCR for *Pneumocystis jirovecii* in BAL	6 (43)
Positive for both immune morphology and PCR in BAL	3 (21)
Other methods (not based on BAL) contributing to diagnosis	3 (21)

a Excluding clinical assessment.

b According to the ‘Revised Definitions of Invasive Fungal Disease from the European Organization for Research and Treatment of Cancer/Invasive Fungal Infections Cooperative Group and the National Institute of Allergy and Infectious Diseases Mycoses Study Group (EORTC/MSG) Consensus Group’.

c Optical density cutoff value 1.0.

d Halo sign and/or air crescent sign.

### Safety

Complications were reported in 20 (13%) of the cases and were mainly mild. No lethal complications and no cases of pneumothorax were observed. Minor bleeding occurred in four (3%) of the subjects.

## Discussion

The clinical value of BAL as a routine investigation in immunocompromised patients with pulmonary infiltrates of unknown etiology has been questioned for several reasons. First, improvements in radiology (i.e. chest CT scanning) and non-invasive microbiological diagnostics performed on blood or sputum may reduce the need for BAL. Second, because of the invasive character of BAL, clinicians may be reluctant to order it in severely ill cancer patients prone to bleeding. Third, a substantial proportion of BAL procedures may result in findings with no or only marginal impact on the clinical management of an individual patient. These considerations prompted us retrospectively to study the diagnostic yield of 151 BAL investigations in 133 unique patients treated at our institution for hematological malignancies over a 10-year period.

Our main finding was that BAL had a direct impact on clinical management (i.e. altered antimicrobial therapy) in 25% of all cases, either by establishing or contributing to a specific etiological microbiological diagnosis or by leading to cessation of ongoing empirical antibacterial or antifungal therapy. The findings are consistent with those of previous studies (1,5–7,9–11). Our results support BAL as an important diagnostic method also in the future in patients with hematological malignancies with fever and pulmonary infiltrates of unknown etiology.

The diagnostic yield and clinical utility of BAL depend, among other factors, on the patient population. In our patient cohort acute leukemia was the most frequent diagnosis, and about one-third of the patients had undergone allo-HSCT. Therefore, we had two high-risk groups for opportunistic pulmonary infection with a higher likelihood of positive findings from BAL. An overall mortality rate of 59% at the end of the 10-year investigation reflects a severely ill population. About one-third of the patient population were neutropenic, and the majority had high MASCC scores, indicating high vulnerability for infectious complications (17).

Importantly, BAL contributed to the diagnosis in two-thirds of the patients with IPA, and, in those diagnosed with PJP, BAL added diagnostic information in all but one case. The morbidity and mortality of IFD, especially if caused by *Aspergillus* spp. or *Pneumocystis jirovecii*, are high, and early diagnosis together with prompt initiation of antifungal therapy are critical for a favorable outcome (1,2,18–20). Several non-invasive culture-independent methods for diagnosing IFD are available. GM has high sensitivity, and PCR arrays for *Aspergillus* spp. (yielding a faster result) are under development (2,19,21,22). BG, a cell wall component of most fungal species including *Pneumocystis jirovecii*, can be detected in blood for many different IFDs. Its usefulness is limited by low sensitivity, but a negative test is valuable for excluding PJP (23). Diagnosing IFD remains a challenge (4,19,24), but BAL is still a valuable tool in diagnosing this lethal condition in immunocompromised patients.

The low incidence of verified bacterial pneumonia might be attributable to ongoing treatment with empirical or pre-emptive broad-spectrum antibiotics in two-thirds of the cases. This suggests that it is crucial to perform BAL at early onset of symptoms to render meaningful results (3,9). The low incidence of viral pneumonia in our material could be because non-invasive methods, such as PCR for respiratory viruses on nasopharyngeal secretion, are used instead of BAL when this diagnosis is suspected.

Rather than an individualized sampling schedule, we used a standardized procedure with extensive microbiological testing. An important finding of our study is that several BAL analyses yielded no diagnostic information. For example, PSB is used routinely to avoid contamination (1). In the present study, samples from PSB (obtained in 97% of cases), yielded additional information in only one single case. This is consistent with findings reported by Boersma et al. (1). Moreover, the diagnostic impact varied greatly between analyses; a positive result for *Pneumocystis jirovecii* (immune morphology) and Aspergillus (GM) always contributed to the diagnosis of the infectious episode, whereas Candida PCR, for example, was positive in 12 (20%) of the cases, but never led to any change of diagnosis or treatment. Clearly, the usefulness and cost effectiveness of BAL-related procedures and tests need to be regularly re-evaluated. No serious procedure-related complications were observed in the present study, which accords with previous reports (1,3,7,9,12,13). Interestingly, no major bleeding complications occurred, although many patients had severe thrombocytopenia. The practice of giving prophylactic platelet transfusions prior to BAL in severely thrombocytopenic patients has never been proven to be necessary, but continues to seem reasonable.

Our study has some limitations. Reflecting the retrospective nature and complexity of infection diagnostics in these patients (4,19), documentation in patient records was not always clear, making estimates of ‘clinical impact’ difficult. The absence of more precise criteria for performing BAL may have affected patient selection. Our standard protocol for microbiological sampling also underwent moderate changes during the study period. Nevertheless, the study describes real-life data from a well-defined Nordic cohort of consecutive patients undergoing BAL using a well-standardized procedure including extensive microbiological sampling.

We conclude that BAL still contributes to either verifying or excluding some of the important respiratory tract infections in patients with hematological malignancies, in particular IPA and PJP. Standardized procedures for BAL sampling should be continuously revised to exclude unnecessary microbiological tests. Improvements of non-invasive microbiological and radiological methods may reduce the need for performing BAL in the future.

## Appendix

Appendix 1. Bronchoalveolar lavage, standard operating procedure, updated 2012—recommended analyses

**Table:**

*All hematology patients:*
• General culture (quantitative, aerobe, anaerobe)
• Fungal culture, direct fungal microscopy
• Respiratory virus PCR (RS-, adeno-, para influenza-, influenza A and B)^a^
• Respiratory virus immunofluorescence (RS-, adeno-, para influenza-, influenza A and B)^b^
• Legionella PCR (cultivation is performed automatically in case of positive PCR)^c^
• Legionella immunofluorescence^d^
• Protected specimen brush: general culture, fungal culture, Legionella PCR (culture is performed automatically in case of positive PCR)^c^
• *Pneumocystis jirovecii* immunofluorescence (PCR is performed automatically in case of negative result)
• Mycobacteria direct microscopy and general culture
• General virus culture
• Papanicolaou cytology
*Immunocompromised patients:*
• Cytomegalovirus PCR
• Herpes simplex type 1 and 2 PCR^c^
• Aspergillus antigen and Aspergillus PCR^c^
*In selected cases:*
• Pneumococci PCR^c^
• Mycoplasma + Chlamydophilia pneumonia PCR
• *M. tuberculosis* complex PCR^c^
• Cytomegalovirus Antigen^b^

a Added 2012.

b Excluded 2012.

c Added 2007.

d Excluded 2007.
